# Comparison of a Robotic and Patient-Mounted Device for CT-Guided Needle Placement: A Phantom Study

**DOI:** 10.3390/jcm11133746

**Published:** 2022-06-28

**Authors:** Yannick Scharll, Alexander Mitteregger, Gregor Laimer, Christoph Schwabl, Peter Schullian, Reto Bale

**Affiliations:** Department of Radiology, Section of Interventional Oncology—Microinvasive Therapy (SIP), Medical University of Innsbruck, Anichstr. 35, 6020 Innsbruck, Austria; yannick.scharll@i-med.ac.at (Y.S.); alexander.mitteregger@student.i-med.ac.at (A.M.); gregor.laimer@i-med.ac.at (G.L.); christoph.schwabl@i-med.ac.at (C.S.); peter.schullian@i-med.ac.at (P.S.)

**Keywords:** percutaneous, robotic, radiofrequency ablation, phantom study, accuracy evaluation

## Abstract

Background: Robotic-based guidance systems are becoming increasingly capable of assisting in needle placement during interventional procedures. Despite these technical advances, less sophisticated low-cost guidance devices promise to enhance puncture accuracy compared with the traditional freehand technique. Purpose: To compare the in vitro accuracy and feasibility of two different aiming devices for computed-tomography (CT)-guided punctures. Methods: A total of 560 CT-guided punctures were performed by using either a robotic (Perfint Healthcare: Maxio) or a novel low-cost patient-mounted system (Medical Templates AG: Puncture Cube System [PCS]) for the placement of Kirschner wires in a plexiglass phantom with different slice thicknesses. Needle placement accuracy as well as procedural time were assessed. The Euclidean (ED) and normal distances (ND) were calculated at the entry and target point. Results: Using the robotic device, the ND at the target for 1.25 mm, 2.5 mm, 3.75 mm and 5 mm slice thickness were 1.28 mm (SD ± 0.79), 1.25 mm (SD ± 0.81), 1.35 mm (SD ± 1.00) and 1.35 mm (SD ± 1.03). Using the PCS, the ND at the target for 1 mm, 3 mm and 5 mm slices were 3.84 mm (SD ± 1.75), 4.41 mm (SD ± 2.31) and 4.41 mm (SD ± 2.11), respectively. With all comparable slice thicknesses, the robotic device was significantly more accurate compared to the low-cost device (*p* < 0.001). Needle placement with the PCS resulted in lower intervention time (mean, 158.83 s [SD ± 23.38] vs. 225.67 s [SD ± 17.2]). Conclusion: Although the robotic device provided more accurate results, both guidance systems showed acceptable results and may be helpful for interventions in difficult anatomical regions and for those requiring complex multi-angle trajectories.

## 1. Introduction

New sophisticated tools are increasingly developed for diagnostic and therapeutic percutaneous interventions, with a wide range of applications, such as biopsy and thermal ablation [1,2,3,4]. Navigation software and robotic assistance may enhance puncture accuracy, increase patient safety and reduce radiation exposure as well as procedure duration [5]. Precise planning and accurate needle placement are key for ablative procedures when multiple needles need to be placed at once in order to achieve overlapping ablation zones. By increasing the speed and accuracy of multiple placements, large tumors and multifocal diseases can be treated efficiently [6].

Image-guided needle placement may be based on CT, MRI, ultrasound, and X-ray. MRI provides an excellent soft tissue resolution but it is expensive and requires MRI compatible instruments [7]. In ultrasound, it is not always possible to visualize the tumor adequately due to shadowing artefacts caused by air or bone. Many different interventional procedures are routinely performed in a CT environment, delivering three-dimensional (3D) contrast-enhanced high quality image data. Navigation systems may assist in planning and needle placement, and help to reduce the radiation exposure to clinical staff and patients [8].

A variety of guidance and navigation systems have been developed using active systems with visual or electromagnetic tracking. The robotic approach offers several advantages over other assistance systems. No direct line of sight is needed and the ability to function is unaffected by the presence of ferrous materials [9]. Other passive, patient-mounted needle holders have been developed [10] to improve interventional accuracy taking into consideration a simplified procedure with lower acquisition costs. 

The aim of this study is to compare the accuracy of a commercially available robotic system (Maxio, Perfint Healthcare, Chennai, India) with a low-cost patient-mounted system for CT-guided needle navigation (Puncture Cube System, Medical Templates AG, Egg, Zurich, Switzerland). In addition, the usability, handling time and drawbacks of both techniques are discussed. 

## 2. Materials and Methods

### 2.1. Phantom

**Figure 1:**
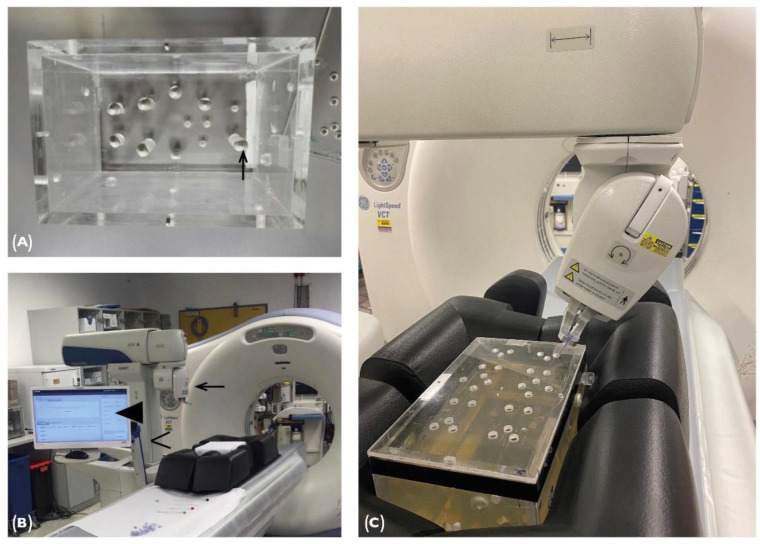
(**A**) The plexiglass phantom (view from above) contains 8 aluminum target points (↑). (**C**) Filled with gelatin, the cover can be mounted in 4 different positions, giving a high variety of entry points. (**B**) The Maxio robot-assisted systemneeds to be docked at the CT table side on a special registration plate. The setup for puncture with the Maxio robot-assisted system contains key components such as the Maxio workstation (◀), the multi-axis electromechanical arm (<) and the end-effector (←).

The phantom used for this study was specifically built for this purpose and was previously used in different studies [11,12,13]. It consists of a plexiglass box (220 mm × 150 mm × 175 mm, Figure 1) with 8 conically-shaped target markers inside the phantom, having aluminum tips with a depth of up to 13 cm. It has a removable cover with 28 holes drilled in it, measuring 8 mm in diameter. By flipping and rotating the lid, 112 individual entrance points can be chosen. The phantom is filled with gelatin, mimicking the consistency of soft tissue. 

### 2.2. Maxio Roboti-assisted Needle Placement and Experimental Set-Up

**Figure 2:**
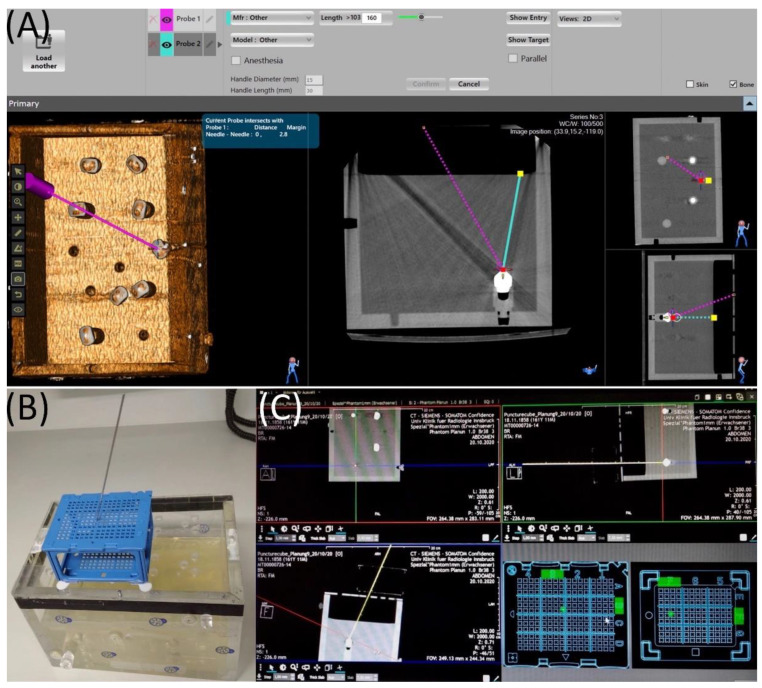
(**A**) Screenshot from the Maxio workstation during the planning procedure of a puncture pathway. Probe 1 (violet path) is confirmed. This picture shows the actual planning of the trajectory of Probe 2 (turquoise line). The red square marks the target point and is already placed on the aluminum tip. The entrance point (yellow square) has to be defined in a next step. All three planes can be used for this purpose. (**B**) The PC is mounted to the phantom. (**C**) In the PCS planning software the target (crosshair) as well as the trajectory of the needle can be freely moved. When a valid needle path has been selected, the corresponding holes given by the software are highlighted in green.

To begin the planning procedure, CT datasets of the gel-filled phantom were acquired in 1.25, 2.5, 3.75 and 5 mm slice thickness in a GE LightSpeed VCT CT System (General Electric Company, Boston, MA, USA). The planning software allows 2D and 3D visualization of the volumetric data (Figure 2A). In total, six needles can be planned simultaneously. The needle length can be chosen from 60 to 250 mm. Needle sizes supported by the device range from 11 to 22 gauge, not including 12 gauge. 

In this study, the target point was set as the aluminum tip inside the phantom. The entry point was defined as the middle of the hole inside the cover. The software automatically provides a hint in case of collisions. The workstation determines the minimum length of the probe required to complete the ablation. After confirming the plan, the system software subsequently instructs the operator to move the CT cradle to a prescribed z-axis location. Thereafter, the robotic arm can be activated and will move automatically to the correct location. In this position the physician can clamp the needle holder and manually insert the ablation probe through the bush in one step. The probe is inserted completely to the end of the bush, considering that the needle length must be defined prior to path planning. After the placement of six needles, a CT scan with 1.25 mm slice thickness was performed to verify the position of the needles. 

### 2.3. Puncture Cube System and Experimental Setup

The “Puncture Cube system” (Medical Templates AG, Egg, Zurich, Switzerland) combines a planning software with a single use, sterile guidance device, the “Puncture Cube” (PC). It uses the novel concept of having an external cube with pre-defined holes (highlighted by the software) on two layers to provide a range of different trajectories that can be planned in advance with a virtual needle in the CT images.

The PC consists of a plastic cubic frame with an upper and a lower template, each of them divided into multiple fields alphanumerically marked. Every field is divided into multiple holes. Furthermore, each hole has four corners allowing 768 needle positioning points on the upper template, and 576 on the lower one. The system is plugged together and can be disassembled during a puncture in case the needle is too short. A pre-mounted flexible piece of tape on every foot comfortably enables the possibility of mounting the PC on the patient or in our case on the cover of the phantom. 

After the PC is placed over the target region, the planning procedure can begin. CT datasets of the gel-filled phantom were acquired in 1, 3 and 5 mm slice thickness in a Siemens Somatom Sensation Open CT system (Siemens AG, Erlangen, Germany) and transferred to the dedicated software “Synedra View Professional” (Synedra information technologies GmbH, Innsbruck, Austria). The software detects the cube automatically in the planning scan, overlaying it with a same shaped and identically-sized virtual cube. The virtual cube can also be manually superimposed over the scanned PC with the help of different marks on the templates. After successful registration, the needle path can be planned by dragging a “virtual needle” on standard MPR views in coronal, sagittal and axial view. When the chosen trajectory covers the template, the software automatically identifies and highlights the most suitable hole with its corner in order to successfully reach the selected target (Figure 2B,C). The software provides information about the insertion depth, being implemented using distance indicators on the needles. 

In a final step, the operator moves the needle through the holes highlighted by the software. Finally, a CT scan with 1 mm slice thickness was performed to verify the position of the needles. 

### 2.4. Evaluation

**Figure 3:**
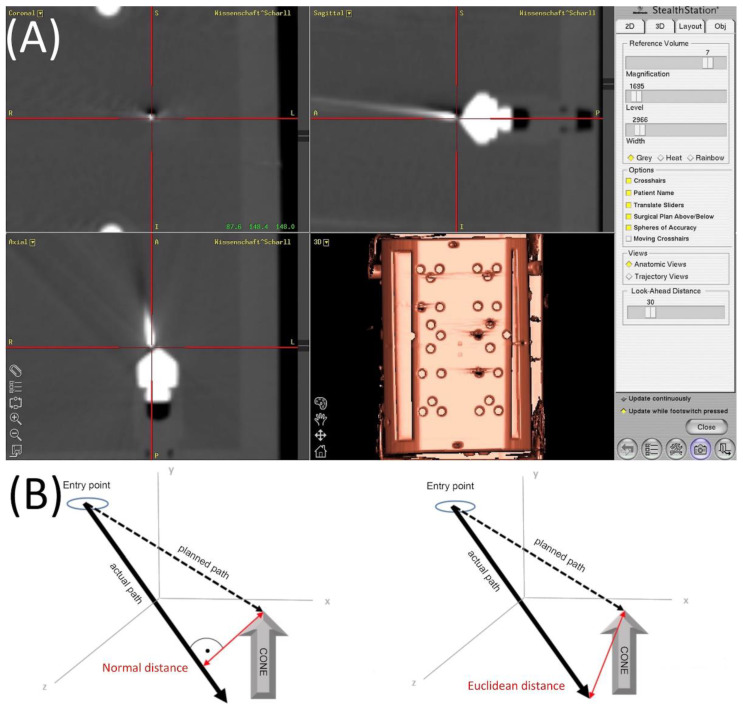
(**A**) Screenshot of the Medtronic Stealth Station Treon to evaluate the needle positioning accuracy with the control CT-data. The x, y and z coordinates (displayed in the left upper quadrant) were measured by positioning the crosshairs in all three planes. The data was then transferred to an Excel spreadsheet for the calculation of the Euclidean and the lateral positioning error. (**B**) The Euclidean distance determines the gap between two points in a multidimensional space. It is calculated using the coordinates of the actual position of the needle tip and the target point, indicating the deviation in the direction of the needle placement. The normal distance describes the shortest possible distance between a point and a straight line.

The post-procedural control CT scans were transferred to the Medtronic Stealth Station Treon via the hospital’s own intranet. For every probe, the “Mach Cranial” software of the station allows the operator to determine the coordinates of the entry point and planned entry point, as well as the tip of the needle and tip of the cone (Figure 3A).

The distances were measured by the calculation of the Euclidean distance (ED) between the target and the positioned wire tip and by the normal distance (ND) between the target and the wire axis (Figure 3B). The definitions are basic formulas in analytical geometry [14]. 

All statistical analyses were performed using SPSS Version 22 (SPSS Inc., Chicago, IL, USA). The distribution of error measurements was graphically checked with histograms and assessed with the Kolmogorov–Smirnov test. Mean errors, standard deviation and maximal values as well as minimal values were calculated. Differences between the device and slice thicknesses were assessed using the Mann–Whitney U test or Independent T-Test, as appropriate. A *p* value of < 0.05 was considered statistically significant.

## 3. Results

In total 560 punctures were performed: 320 punctures were assessed using the Maxio robotic system, including 80 punctures for 1.25, 2.5, 3.75 and 5 mm slice thickness, respectively; 240 needles were placed using the PCS including 80 punctures for 1, 3 and 5 mm slice thickness, respectively. Results are expressed as mean ± SD. 

### 3.1. Maxio

The achieved accuracies measured with the normal and Euclidean distance are summarized in Table 1.

The Kolmogorov–Smirnov Test showed no normal distribution. With histograms, this could be verified, showing mostly right-skewed data for the ED and ND with some outliers.

Using the Mann–Whitney U test, no significant differences between accuracy were observed, comparing all slice thicknesses. 

ED: 1.25 mm vs. 2.5 mm, *p* = 0.56; 1.25 mm vs. 3.75 mm, *p* = 0.71; 1.25 mm vs. 5 mm, *p* = 0.44. 

ND: 1.25 mm vs. 2.5 mm, *p* = 0.57; 1.25 mm vs. 3.75 mm, *p* = 0.81; 1.25 mm vs. 5 mm, *p* = 0.79. 

### 3.2. PCS

The achieved accuracies measured with the normal and Euclidean distance are summarized in Table 2.

The Kolmogorov–Smirnov test showed normal distribution. Using Student’s *t*-test, no significant differences between accuracy were observed, comparing all slice thicknesses. 

ED: 1 mm vs. 3 mm, *p* = 0.13; 1 mm vs. 5 mm, *p* = 0.11; 3 mm vs. 5 mm, *p* = 0.98. 

ND: 1 mm vs. 3 mm, *p* = 0.08; 1 mm vs. 5 mm, *p* = 0.06; 3 mm vs. 5 mm, *p* = 0.99. 

### 3.3. Maxio vs. PCS

**Figure 4:**
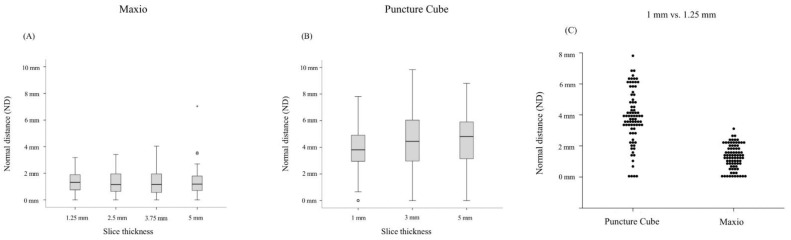
(**A**,**B**) Boxplot of the PCS vs. Maxio comparing the accuracy between all slice thicknesses. (**C**) Dot-plot of the PCS vs. Maxio comparing accuracy for the finest slice thickness.

Comparing both systems using the Mann–Whitney U test, we observed a significant difference between accuracy for all slice thicknesses (Figure 4). 

ED (Maxio vs. PCS): 1.25 mm vs. 1 mm, *p* < 0.001; 2.5 mm vs. 3 mm, *p* < 0.001; 3.75 mm vs. 3 mm, *p* < 0.001; 5 mm vs. 5 mm; *p* <0.001.

ND (Maxio vs. PCS): 1.25 mm vs. 1 mm, *p* < 0.001; 2.5 mm vs. 3 mm, *p* < 0.001; 3.75 mm vs. 3 mm, *p* < 0.001; 5 mm vs. 5 mm, *p* < 0.001.

The PCS punctures required about 30% less time (mean 158.63 s [SD ± 23.38] vs. 225.67 s [SD ± 17.2]). Additional time is needed to physically move and dock the robotic device to the CT tableside, taking approximately 3–4 min [15]. It required approximately the same amount of time to switch off the device, undock and move it to the parking location.

## 4. Discussion

In total, all 560 punctures were performed successfully. Our results of a phantom puncture series show that the Maxio yields greater accuracy compared with the PCS and other image-guided intervention systems. On the other hand, punctures with the PCS required less time. 

The ND might be the most reliable indicator of accuracy. In fact, the aluminum tips prevent the needle from moving forward once they are accurately placed. On the other hand, when not touching the cone, the needle could tend to slip slightly through the gelatin-filled phantom, while the phantom is moved. Fortunately, this did not occur in our experimental setup but could be the case in a millimeter/submillimeter range, even if the needles were embedded tightly in a rigid gel environment. 

It is arguably difficult to compare the results of our two systems with those of existing studies, as different setups were used, and different endpoints were evaluated. 

Our group used the identical phantom with different navigation systems (Table 3). As far as technically possible, we applied the same method to ensure comparability. As an example, Stoffner et al. [11] used a robotic assistance system, the Innomotion, showing accurate results comparable to the Maxio. Moreover, Putzer et al. [13] investigated the accuracy of two electromagnetic navigation systems, the AxiEM and PercuNav, with comparable results to the less complex PCS. 

Overall, the Maxio turned out to be the most accurate guidance system tested by our group. 

Looking at the data for the PCS, there is no significant difference between accuracy, comparing all three slice thicknesses. The difference of the mean value does not exceed 0.5 mm for the ED and 0.6 mm for the ND.

Mokry et al. [16] came to the result that the PCS improves accuracy and reduces intervention time compared to the free-hand method. Their phantom study showed almost equal results compared with ours (3.4 mm ± 2.3 mm vs. 3.84 mm ± 1.75 mm).

Our results show that the precision to arrive at targets decreases with greater target depth. In fact, the modular construction and the plastic material have a negative impact on its stiffness, increasingly affecting accuracy with longer needle paths. 

**Figure 5:**
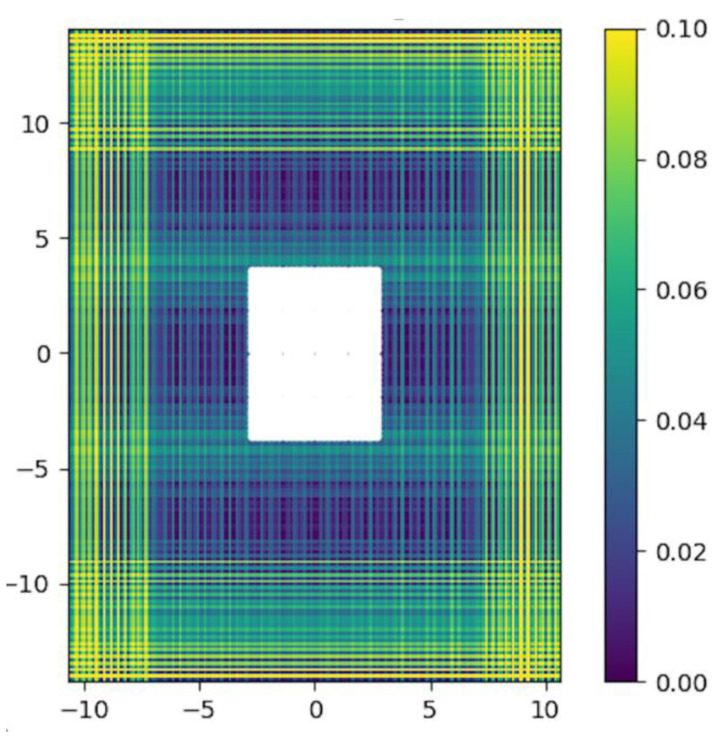
The distribution of errors is color coded graphically for a chosen depth of 10 cm and an area of 400%. The white square in the center corresponds to the surface of the PC.

In contrast to the Maxio, the precision to arrive at the target with the PCS depends on its depth due to a limitation of adjacent valid combinations with the templates. For a chosen depth of 10 cm, the distribution of errors can be color coded graphically (Figure 5), showing a decrease in valid combination in the periphery. 

In a clinical routine, it is important to attach the PC to the patient skin with approximate prior knowledge of the target. The small size of the PCS and its limited range due to a prescribed number of combinations make it crucial to find the right spot. Bony landmarks, prior examinations or topogram images can help to position the device. On the other hand, uneven anatomic locations can cause difficulties attaching the PC due to its flat template. Once the PC is mounted, patient movement will have less impact compared with the Maxio. 

The PCS is faster to use. Taking into account the time the Maxio needs to dock and undock, the PCS allows needle placement considerably faster. Furthermore, the PCS allows the operator to plan and position the probes simultaneously when two physicians are present, making it possible to gain speed. The robotic positioning process of the Maxio is instead fully automatic and does not depend on the experience of the physician. 

Looking at the data for the Maxio, no significant difference can be observed when comparing all four different slice thicknesses. The mean ED and ND show highly accurate results from 1.25 to 5 mm slice thickness. Comparing all slice thicknesses, the difference of the mean value does not exceed 0.25 mm for the ED and 0.1 mm for the ND. 

A cadaver study was performed by Croissant et al. [17], revealing highly accurate results using the Maxio equipment during spinal interventions. The robot-assisted placement of 24 K-wires showed a mean deviation of 1.2 mm in the horizontal-axis and a mean deviation of 0.5 mm in the vertical-axis, comparable with our findings. 

Comparatively, the robotic device did not show any disadvantages in accuracy for a higher insertion depth or a particular angulation. The robotic arm is capable of moving freely within 5 degrees of freedom. It has a large range of achievable orbital needle angles, ranging from −95° to 95° in both lateral and craniocaudal directions. In comparison, the grid of the PCS limits oblique needle insertion to approximately 45°. The Maxio is capable of moving its arm independently, parallel to the CT cradle with a range of 180 mm and perpendicularly 300 mm from the gantry center line to the opposite side of the docked device. 

The software of the Maxio is limited to planning up to six needles simultaneously, which is a disadvantage for ablation of larger tumors or multifocal disease. Larger, more perfused tumors need multi-probe needle placement. A single-pass needle insertion is not the standard procedure for complex clinical needle placements, such as stereotactic radiofrequency ablation of liver tumors. 

Once docked, the Maxio limits physical access on the mounted side of the CT table. This downside is similar to other guidance devices, although the Maxio is quite large, with dimensions of 85 cm × 80 cm × 180 cm, when docked. The quite unwieldy end-effector of the Maxio makes it impossible to plan multiple needle insertions in a small entrance area due to collision. On the other hand, the PCS allows the placement of a considerably higher number of probes simultaneously yet is limited by the small design of the template and the number of holes.

The Maxio allows the operator to manually correct all six needle positions after placement. The PCS also offers the possibility of adjusting the position of the needle as the upper template can be brought in contact to the lower plate. However, this is not possible when more than one needle is obliquely embedded inside the template. 

The planning process is simple on both systems and can be operated by one person. The workstation of the Maxio allows the operator to define the target and entry points, which subsequently executes the planned trajectory, without requiring a physician’s calculation of entry-to-target distance and angulation. In comparison, the PCS only allows single-probe access per treatment plan.

Compared to conventional puncture techniques and low-cost systems, robotic systems have much higher capital costs. The acquisition costs for the PCS are considerably lower compared to the Maxio.

Radiation exposure to the physician can be virtually eliminated with both systems. Conventional needle placement using CT fluoroscopy or incremental needle advancement, needs multiple scans for each probe. Both systems allow several probes to be placed simultaneously. Only a planning scan and a verification scan are generally required, reducing radiation exposure to the patient. Furthermore, the systems yield comparable accuracy using bigger slice thickness, further reducing radiation exposure. On the other hand, planning on a 5 mm CT data set turned out to be more time consuming. The lower spatial resolution made it harder to locate our target points. All three planes had to be examined to estimate the exact spots.

Some limitations are worth mentioning due to the ex vivo environment. A limitation of this phantom study is the inability to consider respiratory movement. Input errors, due to respiratory motion, are key points in the clinical setting. Different techniques beyond active or passive movement could not be applied during this phantom study. Gating the patients’ breathing is a key component of normal clinical routine.

Unlike the flat cover of the phantom, uneven human body surfaces could cause difficulties attaching the PCS. In a clinical routine, inaccuracies might even increase due to the movement of the skin when placed on a patient. Gelatin, which is the most commonly-used phantom material in the literature, shows similar behavior to human soft tissue. Nevertheless, the living body consists of many kinds of tissue components. Therefore, tissue may be movable especially at the interface of the tissues, which may cause needle bending and target movement during needle insertion. 

## 5. Conclusions

In conclusion, the lower-cost system appears to achieve slightly (though statistically significant) lower accuracy than the higher-cost robotic system. Punctures with the Maxio are more accurate but more time consuming compared to the more affordable PCS. No significant loss in accuracy can be observed performing the planning scans in 1 to 5 mm slice thickness, reducing radiation exposure. Both navigation systems work reliably and may be easily included in the clinical routine. Nevertheless, both systems reach their limits for interventions needing multi-probe placements such as stereotactic radiofrequency ablation of multifocal liver tumors. Furthermore, the accuracy required depends on different clinical applications. The minimum required safety margin for certain tumors (i.e., HCC and colorectal liver metastasis) is >5 mm [18,19]. Therefore, using the Maxio robot the mismatch of 1 mm is negligible. However, mean deviations of 4 mm using the PCS may be insufficient for this purpose. Nonetheless, both guidance systems may play a promising role for interventions with difficult anatomical conditions and complex multi-angle trajectories. 

## Figures and Tables

**Table 1:** Normal distance and euclidean distance under guidance with the Maxio.

	ND				ED			
Slice Thickness	Mean	SD	Min	Max	Mean	SD	Min	Max
1.25 mm	1.28	0.79	0	3.18	1.50	0.87	0	3.25
2.5 mm	1.25	0.81	0	3.42	1.55	1.18	0	6.69
3.75 mm	1.35	0.99	0	4.04	1.61	1.30	0	6.28
5 mm	1.35	1.03	0	7.03	1.73	1.19	0	7.14

**Table 2:** Normal distance and euclidean distance under guidance with the PCS.

	ND				ED			
Slice Thickness	Mean	SD	Min	Max	Mean	SD	Min	Max
1 mm	3.84	1.75	0	7.82	4.14	1.70	0	7.99
3 mm	4.41	2.31	0	9.83	4.62	2.25	0	9.89
5 mm	4.41	2.11	0	8.80	4.61	2.07	0	8.85

**Table 3:** Comparison of the accuracy of the Maxio and PCS to the previously reported results of our group. Venturi et al. [12] investigated the accuracy of the ArciNav patient-specific template based guidance system; Stoffner et al. [11] used the Stealth Station Treon optical navigation system in combination with an aiming device and the Innomotion robot; and Putzer et al. [13] tested the AxiEM and the PercuNav electromagnetic navigation systems.

		ND	ED
**Maxio**	Mean and SD (mm)	1.28 (±0.79)	1.50 (±0.87)
	Range (mm)	3.18	3.25
**PCS**	Mean and SD (mm)	3.84 (±1.75)	4.14 (±1.70)
	Range (mm)	7.82	7.99
**ArciNAV**	Mean and SD (mm)	1.42 (±0.66)	2.52 (±0.64)
	Range (mm)	1.33	3.94
**Stealth Station Treon**	Mean and SD (mm)	1.64 (±0.92)	1.94 (±0.91)
	Range (mm)	4.57	4.79
**Innomotion**	Mean and SD (mm)	1.42 (±0.78)	1.69 (±1.42)
	Range (mm)	2.89	2.72
**AxiEM**	Mean and SD (mm)	3.29 (±1.51)	3.86 (±2.28)
	Range (mm)	9.61	14.70
**PercuNav**	Mean and SD (mm)	3.76 (±1.59)	4.42 (±1.33)
	Range (mm)	7.43	6.26
